# How Good is Stratification and Prediction Model Analysis Between Primary and Revisional Roux-en-Y Gastric Bypass Surgery? A Multi-center Study and Narrative Review

**DOI:** 10.1007/s11695-023-06532-3

**Published:** 2023-03-11

**Authors:** Mohamed Hany, Ahmed Zidan, Karim Sabry, Mohamed Ibrahim, Ann Samy Shafiq Agayby, Moustafa R. Aboelsoud, Bart Torensma

**Affiliations:** 1 Department of Surgery, Medical Research Institute, Alexandria University, 165 Horreya Avenue, Hadara, Alexandria, 21561 Egypt; 2 Bariatric Surgery at Madina Women’s Hospital (IFSO-Certified Bariatric Center), Alexandria, Egypt; 3 Department of Surgery, Ain Shams University, Cairo, Egypt; 4 Leiden University Medical Center (LUMC), Leiden, The Netherlands

**Keywords:** Revision surgery, Prediction model, Stratification, Excess weight loss, Roux-en-Y gastric bypass, Sleeve gastrectomy, Gastric band, Vertical banded gastroplasty

## Abstract

**Introductions:**

Revision surgery because of weight recurrence is performed in 2.5–33% of primary vertical banded gastroplasty (VBG), laparoscopic sleeve gastrectomy (LSG), and gastric band (GB) cases. These cases qualify for revisional Roux-en-Y gastric bypass (RRYGB).

**Methods:**

This retrospective cohort study analyzed data from 2008 to 2019. A stratification analysis and multivariate logistic regression for prediction modeling compared the possibility of sufficient % excess weight loss (%EWL) ≥ 50 or insufficient %EWL < 50 between three different RRYGB procedures, with primary Roux-en-Y gastric bypass (PRYGB) as the control during 2 years of follow-up. A narrative review was conducted to test the presence of prediction models in the literature and their internal and external validity.

**Results:**

A total of 558 patients underwent PRYGB, and 338 underwent RRYGB after VBG, LSG, and GB, and completed 2 years of follow-up. Overall, 32.2% of patients after RRYGB had a sufficient %EWL ≥ 50 after 2 years, compared to 71.3% after PRYGB (*p* ≤ 0.001). The total %EWL after the revision surgeries for VBG, LSG, and GB was 68.5%, 74.2%, and 64.1%, respectively (*p* ≤ 0.001). After correcting for confounding factors, the baseline odds ratio (OR) or sufficient %EWL ≥ 50 after PRYGB, LSG, VBG, and GB was 2.4, 1.45, 0.29, and 0.32, respectively (*p* ≤ 0.001). Age was the only significant variable in the prediction model (*p* = 0.0016). It was impossible to develop a validated model after revision surgery because of the differences between stratification and the prediction model. The narrative review showed only 10.2% presence of validation in the prediction models, and 52.5% had external validation.

**Conclusion:**

Overall, 32.2% of all patients after revisional surgery had a sufficient %EWL ≥ 50 after 2 years, compared to PRYGB. LSG had the best outcome in the revisional surgery group in the sufficient %EWL group and the best outcome in the insufficient %EWL group. The skewness between the prediction model and stratification resulted in a partially non-functional prediction model.

**Graphical Abstract:**

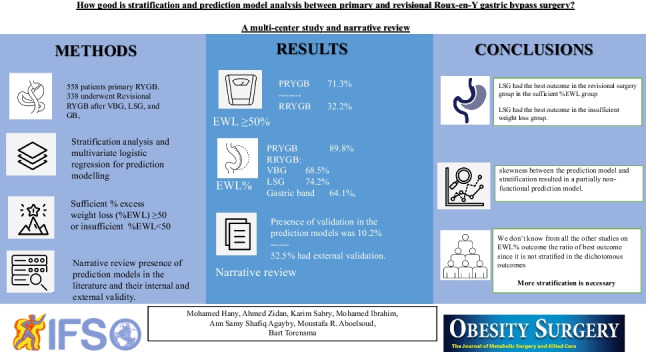

## Introduction

Bariatric metabolic surgery (BMS) is an efficient procedure that can result in considerable sustained weight loss (WL) in patients with obesity, resolve medical problems associated with obesity, and improve quality of life [1]. The long-term health effects are essential for good outcomes in BMS [2, 3]. Unfortunately, weight recurrence occurs between 18 and 24 months after surgery in 30% of patients [4]. The rate of conversion due to insufficient WL or weight recurrence is between 2.5 and 33% after primary vertical banded gastroplasty (VBG), laparoscopic sleeve gastrectomy (LSG), and gastric band (GB) procedures [5–7].

Revisional Roux-en-Y gastric bypass (RRYGB) is commonly employed to revise bariatric procedures because it can effectively manage weight recurrence and obstructive complications [8–12].

Primary Roux-en-Y gastric bypass (PRYGB) and primary VBG, LSG, or GB are different techniques. RYGB is a mixed procedure with restrictive and malabsorptive functions, whereas VBG, LSG, and GB are primarily restrictive procedures. All these procedures also involve changes to the gut hormones and microbiomes [13–15].

Generally, the outcome of results analyses can provide an overestimation or underestimation of the treatment effect. One solution to correct or test for this is stratification analysis, which is defined as sorting data into distinct groups or layers and creating multiple subgroups. Thus, confounding of interest can be defined and corrected, and a more realistic outcome can be presented. Stratification has become an increasingly popular approach [16]. Reflecting this technique on BMS, comparing different surgical techniques and testing the groups within and between each other based on stratified outcomes is more interesting than analyzing different surgeries in the same single revision group.

This study aimed to test how well stratification outcomes compared to unstratified outcome results in a stratified revisional surgery group in combination with testing a prediction model and comparing them with PRYGB as a control to understand the treatment effect and identify the predictive value.

In addition, we tested the presence of prediction models and validation status in the literature by searching for (revisional) BMS models and conducting a narrative review of the presence of prediction models and their associated internal and external validity.

## Methods

This retrospective study on stratification and predictive variables of sufficient %EWL between PRYGB and RRYGB weight recurrence in VBG, GB, and LSG surgery was conducted with patients who completed a 2-year follow-up between 2008 and 2019 at Madina Women’s Hospital and Medical Research Institute, Alexandria University, Alexandria, Egypt, and Ain shams University, Cairo, Egypt. The study was approved by the appropriate ethics committee and was performed in accordance with the ethical standards of the 1964 Declaration of Helsinki. All patients provided informed consent for the data to be used for research publication.

### Patient Selection

Two groups were defined as responders or non-responders to WL after PRYGB and RRYGB according to %EWL ≥ 50 and %EWL < 50, respectively [17].

%EWL was calculated using the formula: %EWL = (initial body weight − current body weight) / (initial body weight − ideal body weight) × 100% (in which ideal body weight is defined by the weight corresponding to a BMI of 25 kg/m^2^).

Percentage total weight loss (%TWL) was calculated using the formula: %TWL = ((initial body weight − current body weight) / initial body weight) × 100%.

### Study Endpoints

First, testing the differences in stratification and unstratified outcome results in different revisional surgery groups. In addition, prediction models were built and tested using the stratified outcomes. All were compared with PRYGB as the control to understand the treatment effect and identify predictive values.

Second, conducting a narrative review of the presence of prediction models and validation status in the BMS field literature.

### Data Collection

The analyzed data included demographic characteristics and associated medical conditions, laboratory investigations, preoperative workups, postoperative follow-ups, and time between surgeries.

Body mass index (BMI), nadir BMI, pre-revision BMI, %EWL, %TWL, percentage of excess BMI loss (%EBMIL) after primary surgery, BMI 3 months post-revision in the RRYGB group, as well as BMI, %EWL, %TWL, and %EBMIL 2 years after surgery for PRYGB were measured in the RRYGB group.

#### Surgical Technique

RRYGB and PRYGB surgeries were performed by two independent surgeons (who operate on approximately 800 patients/year) and four assistant surgeons, per the standard protocols and international guidelines. For PRYGB and RRYGB, the lengths of the biliopancreatic and alimentary limbs were 100 cm each (Appendix 1).

#### Statistical Analysis

Descriptive and inferential statistics were used for the analyses. All data were tested for normality using the Kolmogorov − Smirnov test, Q-Q plot, and Levene’s test. Categorical variables are expressed as numbers and percentages. Normally and non-normally distributed continuous variables are presented as means and standard deviations (SDs) and medians and interquartile ranges, respectively. When appropriate, categorical variables were tested using Pearson’s chi-square test or Fisher’s exact test. Normally distributed continuous data were tested with dependent samples utilizing Student’s *t*-test for pre and postoperative results. The Wilcoxon signed-rank test was used for skewed (non-parametric) data. For the three RRYGB subgroups and PRYGB, a one-way ANOVA test was performed with multiple Tukey pairwise comparisons between the groups.

Predictors were evaluated and corrected using univariate and multivariate logistic regression analyses. All independent variables, including over 10 events with *P*-values < 0.1, were eligible for multivariate analysis, which was achieved through backward selection. The prediction model was evaluated using a − 2 log-likelihood test. *P*-values < 0.05 were considered statistically significant. Statistical analyses were performed using R-studio (version 4.0.4).

#### Stratification

Multiple stratification strategies were used to identify differences and trends within and between all groups. Two main stratifications of %EWL > 50 were performed, whereby the revisional cohort was stratified into three sub-strata (VBG, LSG, and GB). All were compared within and between each stratification and sub-stratification. Stratification 1 and 2 included patients with %EWL ≥ 50 and %EWL < 50, respectively.

#### Sample Size

G*power version 3.1.9.5 was used for sample size calculation. As this was a retrospective database study, we determined the minimum quantity required to identify the differences in %EWL. To detect a difference of delta 0.1 in the %EWL between PRYGB and RRYGB with an alpha of 0.05 and a beta > 0.8, we needed 51 patients per arm. Thus, a minimum of 306 patients, 153 for RRYGB and 153 for PRYGB, were required.

#### Methods for Narrative Review

All relevant and present studies on prediction modeling and internal or external validation in (revisional) BMS were collected for the narrative review.

PubMed was searched from its inception to November 28, 2022. We used the following terms and their synonyms, which were truncated where necessary:

- Prediction/Predictors/Prediction/predictive/predict*/Model/modeling/model*/ AND Validation/validating/valid*/internal/external AND bariatric surgery/ bariatric surger*

Grey literature was also searched, and a reference crosscheck was performed to detect eligible articles that were not identified in the previous search. This search was conducted without restrictions on the language or publication date. The risk of bias for the methodological quality of each included study was not assessed because this narrative review aimed only to identify studies on prediction modeling and validation in BMS and baseline outcomes.

Two reviewers (BT and MH) independently screened the titles and abstracts of the studies based on the inclusion criteria, prediction model, validation study, and BMS. Thereafter, the same reviewers independently reviewed the remaining full-text reports for eligibility.

Furthermore, questions regarding prediction and validation requirements were analyzed and discussed under the purview of this review.

## Results

This retrospective cohort study analyzed data between 2008 and 2019. A total of 558 and 338 patients underwent PRYGB and RRYGB after VBG, LSG, and GB, respectively, and completed 2 years of follow-up.

### Lost to Follow-up Patients

Between 2008 and 2019, 691 patients underwent PRYGB, of whom 558 (80.8%) completed 2 years of follow-up, and 133 (19.2%) were lost to follow-up. In the revisional cohort, 516 patients underwent revision surgery; 97 (18.8%) were excluded because of other revision surgery than RYGB; in total, 338 (65.5%) completed 2 years of follow-up, and 81 (15.7%) were lost to follow-up.

### Unstratified

#### Baseline Characteristics

BMI before primary surgery was 47.6 ± 7.04 and 50.4 ± 8.5 kg/m^2^ (*p* ≤ 0.001) in the PRYGB and RRYGB cohorts, respectively. Pre-revisional surgery, the BMI of 42.4 ± 6.04; %EWL, %TWL, and %EBMIL were 22.1, 11.3, and 14.0%, respectively (Table 1).

**Table 1:** Baseline characteristics of patients in the primary RYGB and revision RYGB groups (non-stratified)

	Primary RYGB (*N* = 558)	Revision RYGB (*N* = 338)	*p* value
Unstratified before RYGB			
Age in years, mean ± SD	37.8 ± 11.1	43.2 ± 7.2	< 0.001
Sex (female), *n* (%)	407 (72.9)	290 (85.8)	0.004
Weight in kg, mean ± SD	133.0 ± 26.1	140.3 ± 26.3	< 0.001
Excess in kg, mean ± SD	68.9 ± 22.3	76.3 ± 24.7	< 0.001
BMI in kg/m^2^, mean ± SD	47.6 ± 7.04	50.4 ± 8.5	< 0.001
Unstratified pre-revision			
Weight mean ± SD	-	117.6 ± 15.7	-
BMI mean ± SD	-	42.4 ± 6.04	-
%EWL median (IQR)	-	22.1 (39.4)	-
%TWL median (IQR)	-	11.3 (30.4)	-
%EBMIL median (IQR)	-	14.0 (25.8)	-
Unstratified weight after 2 years			
Weight in kg, mean ± SD	78.9 ± 20.5	95.4 ± 17.2	< 0.001
BMI in kg/m^2^, mean ± SD	28.5 ± 7.0	34.4 ± 6.5	< 0.001
%EWL median (IQR)	89.8 (52.7)	38.6 (27.6)	< 0.001
%TWL median (IQR)	39.4 (60.1)	19.1 (22.4)	< 0.001
%EBMIL median (IQR)	54.9 (32.4)	22.7 (9.2)	< 0.001
Sufficient and insufficient EWL within the group EWL classes			
%EWL ≥ 50, *n* (%)	398 (78.5)	109 (21.5)	< 0.001
%EWL < 50, *n* (%)	160 (41.1)	229 (58.9)	
Sufficient and insufficient EWL within the group PRYGB and RRYGB			
%EWL ≥ 50, *n* (%)	398 (71.3)	109 (32.2)	< 0.001
%EWL < 50, *n* (%)	160 (28.7)	229 (67.8)	

After 2 years, %EWL and %TWL within the PRYGB cohort were 89.8 ± 52.7 and 39.4 ± 60.1, respectively. For the RRYGB cohort, this was 38.6 ± 27.6 and 19.1 ± 22.4, respectively (*p* = 0.001) (the rest of the baseline characteristics are presented in Table 1).

### Stratified

#### Percentage of Sufficient and Insufficient EWL (≥ 50% and <) Within the Group EWL Classes Between PRYGB and RRYGB

Overall, 78.5% of patients in the PRRYGB group had a sufficient EWL ≥ 50%, compared to 21.5% in the RRYGB.

41.1% in the PRYGB had insufficient (%EWL < 50), compared to 67.8% in the RRYGB.

#### Percentage of Sufficient and Insufficient EWL (≥ 50% and <) Within PRYGB and RRYGB

Overall, 71.3% of patients in the PRYGB group had sufficient EWL (≥ 50%) compared to 28.7% who were insufficient.

In the RRYGB group, 32.2% had sufficient EWL, compared to 67.8% who had insufficient %EWL (< 50%) (*p* < 0.001) (Table 1).

#### Before Revision Surgery

WL, %EWL, %TWL, %EBMIL, and BMI after primary surgery and before revision surgery, stratified in both %EWL classes and revisional surgery groups, were not significantly different (*p* ≥ 0.48).

#### Stratified Characteristics of Sufficient %EWL ≥ 50 (Stratum 1)

Patients who underwent LSG after revision surgery had the best outcomes, compared to VBG and GB (*p* ≤ 0.001). Patients in stratum 1 for PRYGB were significantly younger than those in PRYGB stratum 2 and both RRYGB strata (*p* ≤ 0.0001).

Those who underwent VBG had the greatest number of years between surgery (median [IQR] 10 [3.75]) and LSG, where the shortest with 5 (2.0) years (*p* = 0.001); however, no difference was observed between the p1 and p2 main strata (*p* = 0.452) (Table 2).

**Table 2:** Stratification of the pre and postoperative 3 months, 2-years in %EWL ≥ 50 (strata 1)

After 2 years	Primary	Primary + revision	Primary + revision	Primary + revision	
	RYGB (s1.0)	VBG (s1.1)	Sleeve (s1.2)	Gastric band (s1.3)	*P* value S1.1–1.0 |1.1–1.2 S1.2–1.0 |1.1–1.3 S1.3–1.0 |1.2–1.3
%EWL > 50, n (%)	398 (78.5)	38 (7.5)	48 (9.4)	23 (4.5)	< *0.001*
Preoperative					
Age (years), mean ± SD	32.4 ± 6.9	41.6 ± 9.5	42.5 ± 7.4	42.6 ± 6.9	S1.1–1.0 = 0.001 S1.2–1.0 = 0.001 S1.3–1.0 = 0.001 Rest > 0.83
Gender: Female, N (%)	292 (73.4)	33 (95.7)	42 (91.3)	20 (96.6)	< *0.001*
Years between surgery, median (IQR)	-	10 (3.75)	5 (2.0)	7 (3.0)	S1.1–1.2 = 0.001 S1.1–1.3 = 0.001 S1.2–1.3 = 0.001
BMI-preoperative (kg/m^2^), mean ± SD	47.6 ± 7.2	48.3 ± 8.2	51.7 ± 10.0	49.4 ± 5.9	S1.2–1.0 = 0.002 Rest > 0.74
Nadir weight (kg), mean ± SD	-	92.8 ± 10.3	87.5 ± 11.2	88.6 ± 9.4	S1.1–1.2 = 0.06 Rest ≥ 0.25
Nadir BMI (kg/m^2^), mean ± SD	-	32.2 ± 4.2	30.9 ± 4.3	32.0 ± 4.2	All > 0.31
Weight loss after primary surgery (kg), median (IQR)	62 (28.0)	30 (28.0)	35 (33.0)	30 (33.5)	S1.1–1.0 = 0.001 S1.2–1.0 = 0.001 S1.3–1.0 = 0.001 Rest > 0.88
%EWL before revision, median (IQR)	-	48.3 (24.7)	44.1 (22.5)	44.5 (18.3)	All > 0.76
%TWL before revision, mean ± SD	-	23.2 ± 14.7	23.8 ± 15.1	25.6 ± 13.1	All > 0.71
	RYGB (s1.0) *N* = 398	VBG (s1.1) *N* = 38	Sleeve (s1.2) *N* = 48	Gastric band (s1.3) *N* = 23	*P* value S1.1–1.0 |1.1–1.2 S1.2–1.0 |1.1–1.3 S1.3–1.0 |1.2–1.3
%EBMIL before revision, median (IQR)	-	28.6 (17.1)	29.4 (17.1)	28.2 (14.7)	All > 0.80
BMI before revision (kg/m^2^), mean ± SD	-	36.5 ± 4.2	39.1 ± 4.6	37.7 ± 3.5	S1.1–1.2 = 0.019 Rest > 0.42
3 months postoperative					
BMI after revision (kg/m^2^) (3 months), mean ± SD	-	32.6 ± 3.7	35.2 ± 4.5	33.9 ± 3.1	S1.1–1.2 = 0.008 Rest > 0.35
2 years postoperative					
Weight loss after revision (2 years) (kg), mean ± SD	-	26.4 ± 10.7	34.2 ± 13.1	25.9 ± 8.9	S1.1–1.2 = 0.006 S1.2–1.3 = 0.01 Rest > 0.98
BMI after 2 years(kg/m^2^), mean ± SD	24.6 ± 3.6	27.2 ± 2.2	26.8 ± 1.6	28.3 ± 2.5	S1.1–1.0 = < 0.001 S1.2–1.0 = < 0.001 S 1.3–1.0 = < 0.001 Rest > 0.31
%EWL after 2 years, mean ± SD	93.7 ± 15.5	68.5 ± 16.8	74.2 ± 12.9	64.1 ± 16.1	S1.1–1.0 = < 0.001 S1.2–1.0 = < 0.001 S 1.3–1.0 = < 0.001 S 1.3–1.2 = 0.04 Rest > 0.32
%TWL after 2 years, mean ± SD	47.3 ± 9.8	24.7 ± 7.8	30.4 ± 9.1	24.6 ± 1.2	S1.1–1.0 = < 0.001 S1.2–1.0 = < 0.001 S 1.3–1.0 = < 0.001 S 1.3–1.2 = 0.03 Rest > 0.38
%EBMIL after 2 years	58.5 ± 10.6	32.4 ± 9.5	39.3 ± 11.3	32.3 ± 8.9	S1.1–1.0 = < 0.001 S1.2–1.0 = < 0.001 S 1.3–1.0 = < 0.001 S 1.1–1.2 = 0.01 S1.2–1.3 = 0.04 Rest > 0.99

#### Weight Factors

The %EWL achieved 2 years after the revision surgeries for VBG, LSG, and GB was 68.5, 74.2, and 64.1%, respectively; the %TWL was 24.7, 30.4, and 24.6%, respectively. The LSG group had the highest %EWL and %TWL, which significantly differed from that of GB (*p* = 0.04, 0.03), but not VBG (*p* = 0.32, 0.38).

Compared with PRYGB as the control, the %EWL was 93.7 ± 15.5% and the %TWL was 47.3 ± 9.8% (*p* ≤ 0.001).

The BMI after 2 years remained significantly higher in the RRYGB group compared with the control PRYGB group (27.2, 26.8, and 28.3 kg/m^3^ vs. 24.6 kg/m^3^) (*p* ≤ 0.001).

Patients who underwent VBG had the highest nadir weight after primary surgery (92.8 ± 10.3) compared to the lowest (LSG 87.5 ± 11.2) (*p* = 0.06). Patients who underwent LSG had significantly more WL after revision than those who underwent VBG and GB (34.2 vs. 26.4 and 25.9) (*p* = 0.006, 0.01).

**Fig. 1:**
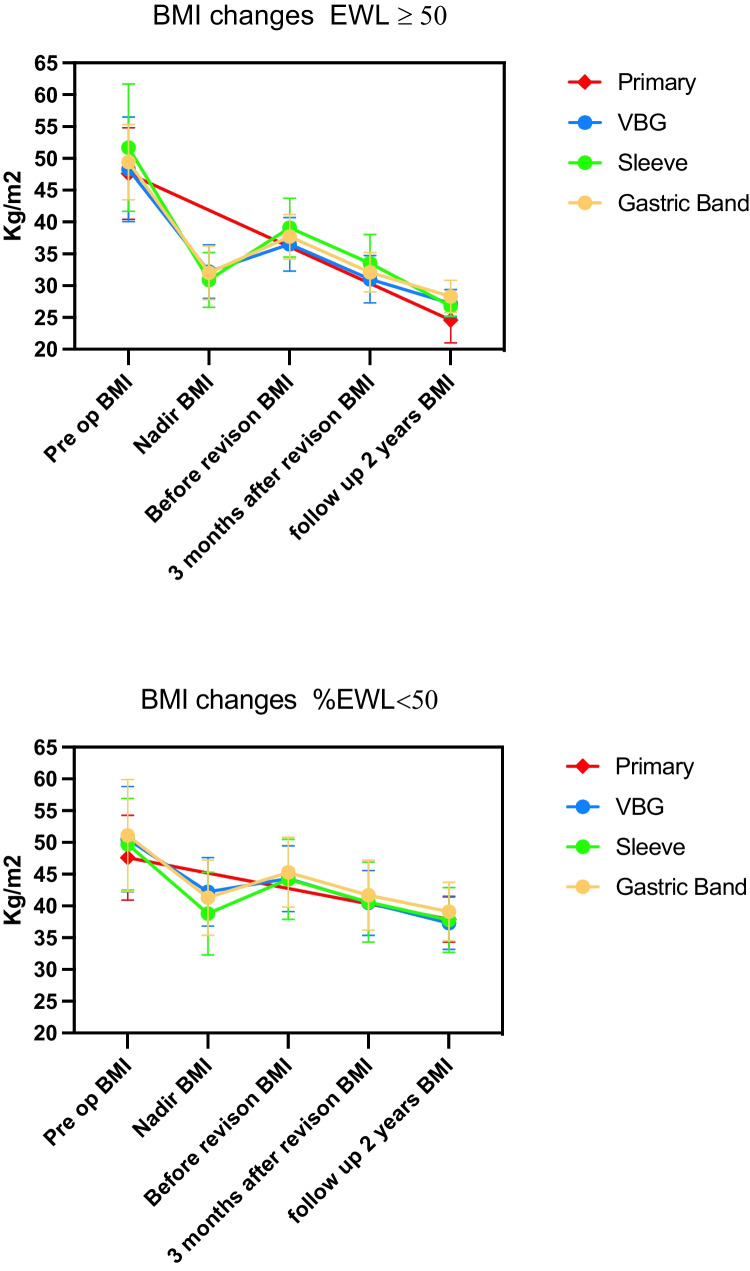
BMI changes in %EWL≥ 50 and %EWL < 50

The lowest nadir weight after the first surgery was associated with the highest %EWL and %EBMIL after 2 years in the RRYGB group (Table 2, Fig. 1).

#### Stratified Characteristics of Insufficient %EWL < 50 (Stratum 2)

The %EWL achieved 2 years after the revision surgeries for VBG, LSG, and GB was 33.3, 33.2, and 28.4% (*p* ≤ 0.001), respectively; the %TWL was 15.7, 15.6, and 13.7%, respectively. Compared with PRYGB as the control, the %EWL was 38.4 ± 8.3% and %TWL was 19.6 ± 5.7% (*p* ≤ 0.001).

Patients who underwent VBG had the most insufficient %EWL < 50 after revision surgery compared to those who underwent GB and LSG (32.9, 17.7, and 8.2%), and combined with GB, had the longest follow-up time between surgeries (*p* ≤ 0.001).

The nadir weight and BMI in the LSG group were significantly lower than in the VBG and GB groups: nadir weight, 105.9 vs. 115.3 and 115.0 kg (*p* = 0.009), respectively.

The PRYGB group had significantly more WL than the RRYGB group after revision surgery (27.25 kg vs. 19.2 kg, 19.0 kg, and 17.3 kg, respectively) (*p* = 0.008). However, PRYGB showed no significant difference in BMI after 2 years compared to RRYGB (Table 3, Fig. 1).

**Table 3:** Stratification of the pre and postoperative 3 months, 2-years in %EWL < 50 (strata 2)

After 2 years EWL < 50%	Primary	Primary + revision	Primary + revision	Primary + revision	*P* value S2.1–2.0 |2.1–2.2 S2.2–2.0 |2.1–2.3 S2.3–2.0 |2.2–2.3
	RYGB (s2.0)	VBG (s2.1)	Sleeve (s2.2)	Gastric band (s2.3)	
	160 (41.1)	128 (32.9)	32 (8.2)	69 (17.7)	< 0.001
Preoperative					
Age (years), mean ± SD	51.5 ± 7.3	43.0 ± 6.4	44.1 ± 7.6	44.3 ± 6.9	S2.1–2.0 = 0.001 S2.2–2.0 = 0.001 S2.3–2.0 = 0.001 Rest > 0.8
Gender: female, n (%)	115 (73.3)	107 (83.6)	31 (96.9)	57 (82.6)	< 0.001
Years between surgery, median (IQR)	-	9 (3)	5(1)	10 (2)	S2.1–2.2 = 0.001 S2.2–2.3 = 0.001 S2.1–2.3 = 0.951
BMI-preoperative kg/m^2^, mean ± SD	47.7 ± 6.7	50.5 ± 8.3	49.7 ± 7.2	51.1 ± 8.8	S2.1–0 = 0.008 S2.3–0 = 0.010 Rest = > 0.8
Nadir weight (kg), mean ± SD	-	115.3 ± 13.5	105.9 ± 19.6	115.0 ± 18.4	S2.1–2.2 = 0.009 S2.2–2.3 = 0.02 Rest = > 0.9
Nadir BMI (kg/m^2^), mean ± SD	-	42.2 ± 5.4	38.8 ± 6.5	41.3 ± 5.9	S2.1–2.2 = 0.009 S2.2–2.3 = 0.07 Rest > 0.8
Weight loss after primary surgery (kg)	27.25 (18.2)	9.5 (19.25)	11.0 (19.0)	10.0 (28.0)	S2.1–2.0 = 0.001 S2.2–2.0 = 0.001 S2.3–2.0 = 0.001 Rest > 0.67
%EWL before revision, median (IQR)	-	13.3 (21.9)	16.5 (18.9)	14.2 (23.1)	All > 0.49
%TWL before revision, mean ± SD	-	8.6 ± 12.5	11.4 ± 12.4	10.4 ± 13.4	All > 0.54
	RYGB (s2.0) *N* = 160	VBG (s2.1) *N* = 128	Sleeve (s2.2) *N* = 32	Gastric band (s2.3) *N* = 69	*P* value S2.1–2.0 |2.1–2.2 S2.2–2.0 |2.1–2.3 S2.3–2.0 |2.2–2.3
%EBMIL before revision, median (IQR)	-	8.6 (14.8)	10.0 (14.3)	8.7 (15.9)	All > 0.49
BMI before revision (kg/m^2^), mean ± SD	-	44.3 ± 5.2	44.2 ± 6.3	45.3 ± 5.5	All > 0.48
3 months postoperative					
BMI after revision (3 months) (kg/m^2^), mean ± SD	-	40.5 ± 5.1	40.6 ± 6.3	41.7 ± 5.5	All > 0.31
2 years postoperative					
Weight loss after revision (kg) (2 years)	-	19.2 ± 6.6	19.0 ± 6.6	17.3 ± 6.8	All > 0.13
BMI after 2 years (kg/m^2^), mean ± SD	37.9 ± 3.6	37.3 ± 4.1	37.8 ± 5.1	39.1 ± 4.6	All > 0.23
%EWL after 2 years, mean ± SD	38.4 ± 8.3	33.3 ± 9.0	33.2 ± 9.1	28.4 ± 9.3	S 2.1–2.0 = < 0.001 S 2.2–2.0 = < 0.001 S 2.3–2.0 = < 0.001 S 2.1–2.3 = 0.001 S 2.2–2.3 = 0.04 Rest > 0.99
%TWL after 2 years, mean ± SD	19.6 ± 5.7	15.7 ± 4.6	15.6 ± 4.2	13.7 ± 4.6	S 2.1–2.0 = < 0.001 S 2.2–2.0 = < 0.001 S 2.3–2.0 = < 0.001 S 2.1–2.3 = 0.001 S 2.2–2.3 = 0.02 Rest > 0.99
%EBMIL after 2 years	24.4 ± 6.4	19.8 ± 5.6	19.7 ± 5.2	17.1 ± 5.6	S 2.1–2.0 = < 0.001 S 2.2–2.0 = < 0.001 S 2.3–2.0 = < 0.001 S 2.1–2.3 = 0.011 S 2.2–2.3 = 0.016 Rest > 0.99

#### Prediction Modeling

The baseline OR for sufficient %EWL ≥ 50 (with estimated LnOdds and *p*-values) were 2.4 (0.928, *p* ≤ 0.001), 1.45 (− 0.523, *p* = 0.003), 0.29 (− 2.143, *p* ≤ 0.001), and 0.32 (− 2.027, *p* = 0.004) for PRYGB, LSG, VBG, and GB, respectively.

Regarding the corrected factors after univariate and multivariate backward selection, age (estimated LnOdds, − 1.192, *p* = 0.0016) was the only significant variable in this study.

After testing all models, all other variables in this study were not significant predictors for %EWL ≥ 50 (*p* ≥ 0.05) (Tables 2 and 3, and Appendices 2, 3, and 4).

### Narrative Review

From the inception of PubMed to November 28, 2022, 41,266 studies on BMS were identified. After applying the search strategy to prediction models and BMS, 672 (1.62%) studies were found (years of publication from 1992 to 2022). After title and abstract (TiAb) selection, 486 studies did not meet the inclusion “prediction model AND bariatric surgery” criteria, and 186 remained (0.45%).

**Fig. 2:**
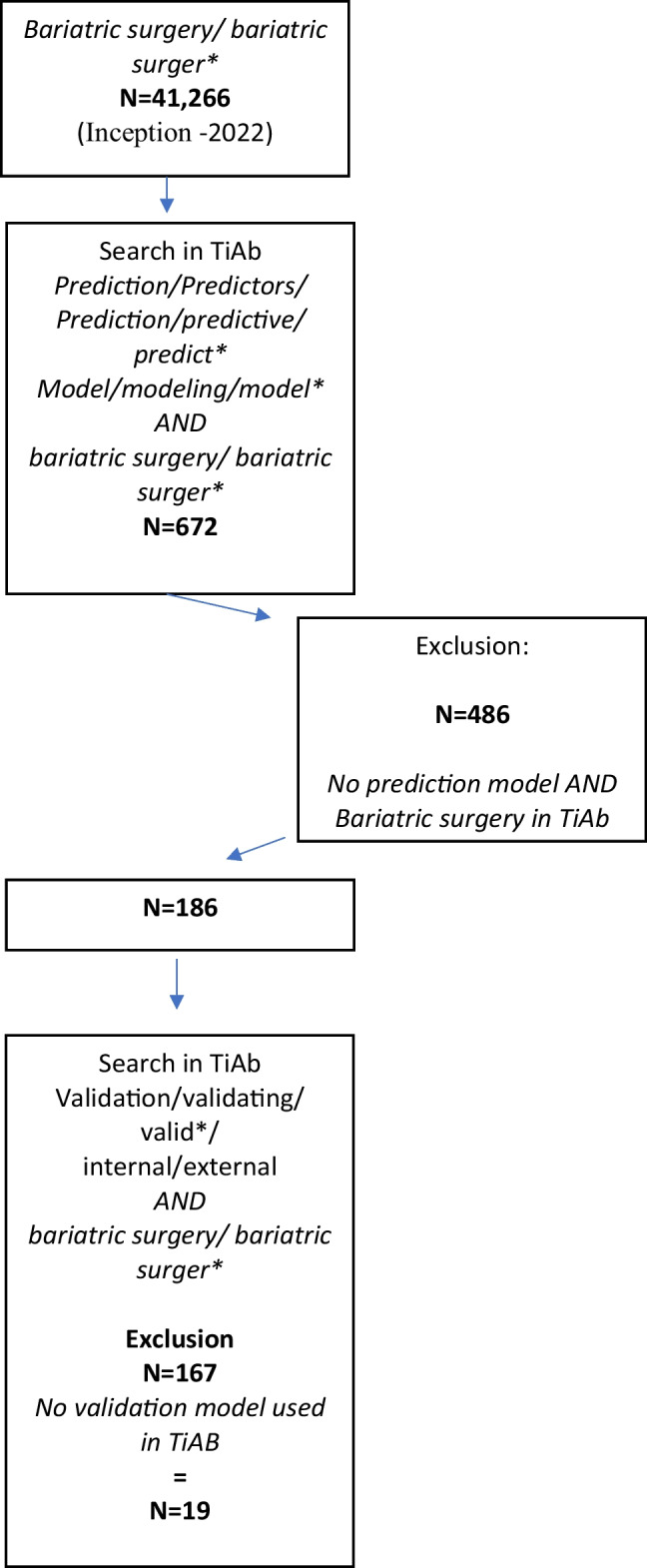
Flow chart

A new TiAb selection was performed for “internal/ external validation” criteria, and 19 articles (0.046% of the 41, 266 BMS studies or 10.2% of the 186 prediction studies) that met this criterion remained (Fig. 2).

Seven studies (36.8%) were published in 2022 [18–24], five (26.3%) in 2021[25–29], one (5.3%) in 2020 [30], two (10.5%) in 2017 [31, 32], and one each (5.3%) in 2015, 2012, 2009, and 2007 [33–36].

#### Validation in Patients with Obesity

In total, 10 (52.6%) studies performed an internal validation [18, 19, 23–26, 28, 32, 34, 36], and 10 (52.6%) performed an external validation [18, 20–22, 27, 29–31, 33, 35] of the prediction model. One study tested internal and external validation. A median (min–max) 760 (70–750, 498) patients were included in the models.

Prediction and validation patients were evaluated once (5.3%) for complication risk, three times (15.8%) for mortality risk, once (5.3%) for gastroesophageal reflux disease (GERD), four times (21.1%) for diabetes remission, once (5.3%) for internal herniation, once (5.3%) for adverse cardiac events, once (5.3%) for frailty scores, twice (10.6%) on the outcome of sufficient and insufficient WL after BMS, three times (15.8%) for WL prediction, once (5.3%) for gastrointestinal leak and venous thromboembolism, and once (5.3%) for BMS quality of life. No studies were found on the prediction and validation of revision BMS.

### Used Variables in the Included Studies

Extraction was conducted on models that presented and predicted variables the most in the validation stage of the studies.

#### Associated Medical Problems

Variables associated with medical problems were utilized for obstructive sleep apnea (OSA) 10 times (52.5%), GERD 10 times (52.5%), hypertension 11 times (57.9%), hyperlipidemia nine times (47.4%), coronary diseases eight times (42.1%), renal problems five times (26.3%), diabetes 14 times (74.7%), liver problems six times (31.6%), arthritis three times (15.7%), cardiac infarct five times (26.3%), and anastomotic leakage three times (15.8%).

#### General Variables

Furthermore, age was included 15 times (78.9%), gender 15 times (78.9%), short-term complication < 30 days once (5.3%), smoking nine times (47.4%), weight 12 times (63.2%), BMI 13 times (68.4%), %EWL five times (26.3%), ethnicity six times (31.6%), and neck, waist circumference, and continuous positive airway pressure/bilevel positive airway pressure (CPAP/BiPAP) once (5.3%).

#### Laboratory Values

High-density lipoproteins were included four times (21.1%), low-density lipoproteins three times (15.8%), triglycerides four times (21.1%), total cholesterol four times (21.1%), fasting blood sugar levels five times (26.3%), and hemoglobin A1C three times (15.8%).

#### Type of Surgery

The types of surgery used were RYGB 16 times (84.2%), LSG 13 times (68.4%), open RYGB twice (10.5%), biliopancreatic diversion with duodenal switch three times (15.7%), single anastomosis duodenal-ileal bypass with sleeve gastrectomy twice (10.5%), and gastric band twice (10.5%).

#### Area Under the Curve (AUC)

In total, 15 studies (78.9%) calculated an AUC for the validation, with an unweighted median (min–max) of the AUC of 0.79 (0.599–0.985) [18–23, 25, 26, 28–32, 34, 36]. The remaining studies used correlation coefficients (*R* or *R*^2^) for all the variables used [24, 27, 33, 35].

## Discussion

This was a retrospective study on stratification that compared unstratified results in created stratified revisional surgery groups, combined with prediction modeling. Additionally, a narrative review of validation and prediction models in the literature was conducted.

BMS has proven effective in achieving significant long-term WL and improving or eliminating associated medical problems [37]. However, only 15–35% of patients reach a desirable %EWL ≥ 50 after surgery [37–40]. To our knowledge, no descriptions are available comparing different stratification analyses with a control group. No studies were found in the narrative review that investigated revision surgery as a model for testing and validating. Our results are consistent with those of other studies. A meta-analysis showed a 20% lower weight reduction after RRYGB than after PRYGB [8].

Insufficient WL is inherent in revisional procedures. The LSG had the best outcome in the sufficient and insufficient groups in our study. Fibrous capsules were found when removing the band, which may cause difficulties when creating a smaller pouch for RYGB. During VBG, scarring was visible through the mesh, and pouch construction was more difficult. Minimal adhesions were visible during LSG; therefore, pouch construction was simpler. Consequently, proper resection is more complex, resulting in a less well-constructed pouch and possibly less WL.

The strength of stratification was clearly visible in this study. First, by comparing the results of the unstratified %EWL and %TWL (as a continuous variable), we confirmed the results for PRYGB and RRYGB [8, 41]. However, when we stratified this (to the dichotomous outcome), the percentage of sufficient (%EWL ≥ 5 0) in PRYGB was only 71.3% compared to 28.7% of insufficient EWL (%EWL < 50). In RRYGB, the sufficient effect was only visible in 32.2% of the cases.

This will initiate debate on when it is unsuitable for stratifying EWL > 50%, EWL < 50%, or any subclassification with an outcome. The results may overestimate “sufficient” in a small group of patients (± 30% was insufficient in PRYGB), which is not achieved by the criteria of EWL50%. Therefore, more stratification, rather than only presenting continuous variables, must be applied. Also, multiple SRs found the outcome in weight loss as continuous %EWL what highlights the misconception that outcomes in the PRYGB and RRYGB possibly have poor responses in sufficient effect. The problem is that we do not know from all the other studies on EWL% outcome the ratio of sufficient effect since it is not stratified in the dichotomous outcomes [31, 42–45], whereby studies define sufficient EWL confirm our findings in both cohorts [25, 46–48].

Furthermore, the focus should be on sufficient and insufficient cut-off criteria and patients who show insufficient effect in both primary and revision surgery.

Several criteria, including weight gain, sufficient, and %EWL > 25%, have been used [49–52]. However, many studies use Reinhold’s criteria [53] for sufficient weight loss > 50%.

We continue to use %EWL > 50 because of the patient effects. Within our profession, the focus remains on the health gain for the patient after BMS. If sufficient WL is achieved at a lower %EWL, the patient may not have achieved sufficient health benefits, such as reducing associated medical problems.

This study showed that 28.7% of PRYGB patients and 67.8% of RRYGB patients did not reach the goal of EWL > 50%. The question remains as to why patients do not respond, even after a second attempt, with a procedure known as the truth-worth procedure. Additionally, why the patient showed insufficient WL on the first attempt? This study showed that with the differences in stratification and prediction models, we could not find any correlations.

### Future of Prediction Models and External Validation

The last decade has witnessed a surge in the development of prognostic prediction models in medicine. A PubMed search by Ramspek et al. showed 84,032 studies on prediction models, of which only 4309 (5%) mentioned external validation in the title or abstract [54]. This narrative review showed that only 10.2% of validation was performed in prediction models. Additionally, only 52.6% of the studies conducted an external validation, consistent with the results of Ramspek et al. [54]. Furthermore, 68.4% of the validation research was conducted in the last 3 years (2022–2020). This reflects that validation studies in the field of BMS are new and should be highlighted, as 95% of prediction models are not externally validated.

Before any prediction model can be implemented, external validation is crucial because prediction models generally perform more poorly in external validation than in the development phase [55]. Ideally, external validation is performed in a separate study by different researchers to prevent adjusting of the model formula based on external validation results [55, 56].

Although it is preferable to have one validated prediction model in all settings and individuals, scientists should strive to validate models in clinically relevant subgroups; as seen in our study, mixed results can occur after the stratification of subgroups.

### Contradictions Between Prediction and Stratification Models in This Study

In the study, it was impossible to create a prediction model that could assist in identifying sufficient WL in patients. However, new contradictions between the prediction and stratification were noted, which can assist us in validating models.

For example, our study showed that preoperative BMI was not a predictor for sufficient %EWL ≥ 50. However, significant differences were observed within and between the stratified PRYGB and RRYGB groups. In the unstratified analysis, there was a significant preoperative BMI difference in favor of PRYGB, although this was not visible in the prediction model.

In our stratified results, we noted that after the primary intervention in the RRYGB group, a higher %EWL or %EBMIL before the revision intervention correlated with higher sufficient WL after 2 years; this was not visible in our prediction model.

The best sufficient WL was observed in the prediction model at younger ages in the PRYGB group compared with the sufficient %EWL ≥ 50 in the RRYGB group. Several other studies found that age is one of the most consistent predictive factors; older patients had poor results [46, 57, 58].

A notable finding was observed within the stratification of insufficient EWL in this study: %EWL < 50 patients in the PRYGB group were significantly older than those in the insufficient RRYGB group (average 51.5 years vs. 42.5 years, respectively). They had significantly higher %EWL and %EBMIL (38.4% vs. an average of 33.2% in the RRYGB *p* ≤ 0.001). The number of years between primary and revisional surgery was not significantly different between the two strata (%EWL < 50 and %EWL > 50). However, there was a significant difference within both strata (sufficient and insufficient EWL) in favor of LSG (5 vs. 8–9 years). LSG showed the best result for predicted sufficiently %EWL within the revision group (OR LSG 1.45 vs. VBG 0.29, *p* ≤ 0.001; GB OR 0.32, p = 0.004).

The prediction model was determined by sufficient EWL, resulting in a model with missed prediction, despite the stratification results favoring a shorter time in both strata as the best sufficient %EWL. Several studies reported that a longer postoperative duration (> 2 years) significantly affected BMS outcomes [58–61].

The results show efficacy when stratified but not when a model is built. Therefore, caution is required when making assumptions based only on a prediction model; this may over or underestimate the outcome.

### What Do We Need to Improve?

Correcting for confounding and bias and creating predictions is always essential; however, we cannot correct or predict what we do not measure.

In the future, external validation and prediction models that provide the entire model formula and specify the prediction horizon with absolute risk calculations are needed to achieve more insight, transparency, and the possibility to develop prediction models. Unfortunately, only 2 of the 19 studies in this narrative review published the intercept or baseline hazard [29], in combination with a beta coefficient [27, 29].

### Limitations

During the analyses, the study’s retrospective design rendered the presentation of all variables a barrier to developing an accurate prediction model. A possible explanation could be that we included PRYGB and RRYGB in one prediction model (since PRYGB was the control and every patient had the chance for a %EWL > 50). One case could have affected the outcomes of the other. Predictive modeling may not be able to test on different data types for datasets and surgery outcomes; this may be tested in the future through external validation. Additionally, the follow-up time in our study cohort was 2 years, which may have been too short of making good predictions. We selected a cut-off of 2 years because it provided the most power for the presented number of patients; with longer follow-ups, the power of the study would decrease for the revisional cases. A longer follow-up could have been used for PRYGB; however, we used the 2-year follow-up to match the outcome. Finally, we conducted a narrative review, but no systematic review was conducted. Narrative reviews aim to identify and summarize what has been published, but no risk of bias assessment was performed, and only data on prediction models were checked. The results from this review will be extended to a systematic review while testing all the prediction studies that did not perform validation.

## Conclusion

Approximately 32.2% of all the patients after revisional surgery had a sufficient %EWL ≥ 50 after 2 years, compared with 71.3% for PRYGB. LSG had the best outcome in the revisional surgery group in the sufficient %EWL group and the best outcome in the insufficient weight loss group. Skewness between the prediction model and stratification resulted in a non-functional prediction model. Extra attention to external validation is necessary to promote the creation of a prediction model that can be generalizable to all patients.

## Data Availability

Data is available at the corresponding author.

## Appendix 1. Surgical workflow per procedure

### Revisional RYGB from VBG

The port sites were modified to avoid the expected areas of adhesions; pneumoperitoneum was created using a visual entry trocar at the left mid-clavicular line approximately 10 cm below the costal margin; this port would be used as a right-hand working port. In cases of adhesions, with the anterior abdominal wall hindering the insertion of the camera and left working ports, the assistant’s port in the left anterior clavicular line was first inserted to dissect these adhesions.

After creating the pneumoperitoneum, adhesions between the stomach and liver were dissected using the energy device Enseal (Ethicon Endo-Surgery, Cincinnati, OH, USA) to identify the mesh to create the correct plane; then, dissection continued up to the angle of His to identify the vertical staple line and expose the gastric serosa. The lesser omentum was dissected off the greater omentum at the body and fundus, with division of the short gastric vessels using the energy device to free the fundus and body. This was followed by dissection of the esophagogastric junction with crural repair using 2/0 V-Loc nonabsorbable sutures (Covidien, Mansfield, MA, USA), if a hiatal hernia was identified. Intraoperative endoscopy was performed at this step to identify the position of the mesh and vertical staple line. Small bowel exploration was attempted before creating the gastric pouch to exclude adhesions or short mesentery to ensure a tension-free gastro-jejunostomy.

The creation of the gastric pouch of RYGB is initiated by opening a window at the lesser curvature 1–2 cm above the mesh, cautiously avoiding injury to the blood supply, followed by horizontal stapling using an Echelon Flex Endopath 60-mm linear stapler (Ethicon Endo-Surgery, Cincinnati, OH, USA) with black reloads. This is continued up to the angle of His using black and green reloads over a 40 Fr bougie, while maintaining the staple line medial to the last vertical staple line inside the VBG pouch. Resection of the gastric fundus and part of the body, including the mesh and the previous staple line, was performed in all cases using the same stapler with green reloads transversely below the mesh.

Biliopancreatic and alimentary limbs lengths were equal, 100 cm each. Gastro-jejunostomy and jejuno-jejunostomy were performed using the same stapler with blue and white reloads, respectively; hand-sewn closure of the gastrostomy and enterostomies were performed using 3/0 V-Loc 180 sutures (Covidien, Mansfield, MA, USA). White reloads were used for dividing the jejunum.

All staple lines were reinforced with continuous seromuscular sutures (3/0 V-Loc 180 (Covidien, Mansfield, MA, USA)). Mesenteric defects were routinely closed using 3/0 V-Loc nonabsorbable sutures (Covidien, Mansfield, MA, USA) in all cases.

Endoscopic examination was repeated to visualize the mucosa of the pouch, and the gastro-jejunostomy was used to assess the vitality of the tissues, exclude intraluminal bleeding, and perform a leak test using air under a water seal. Blue dye leak testing was performed for the gastro-jejunostomy after endoscopy in all cases. A drain was inserted in the left sub-phrenic space at the end of the procedure.

### Revisional RYGB from LSG

Adhesions were dissected around the gastric sleeve using the energy device EnSeal® (Ethicon Endo-Surgery, Cincinnati, OH, USA). The gastric pouch was created from 5 to 6 cm below the esophagogastric junction using an Echelon Flex Endopath 60-mm linear stapler (Ethicon Endo-Surgery, Cincinnati, OH, USA) over a 40 Fr bougie, with gold and blue reloads. The same stapler was used to construct the gastro-jejunostomy and jejuno-jejunostomy using blue and white reloads, respectively, with equal 100 cm biliopancreatic and alimentary limbs. The stapling defects were closed in two layers using barbed sutures (3/0 V-Loc 180 sutures (Covidien, Mansfield, MA, USA)). The staple line in the gastric pouch and remnant stomach was reinforced with seromuscular continuous sutures using the same barbed sutures. All mesenteric defects were closed using 3/0 V-Loc nonabsorbable sutures (Covidien, Mansfield, MA, USA).

### Revisional RYGB from GB

All surgeries in our cohort were performed using a single-step conversion standard: five ports were used, including three 12-mm ports (for the camera and right and left working ports) and two 5-mm ports (for liver retraction and the assistant). A pneumoperitoneum was created after using optical trocars for entry. Adhesions around the stomach were dissected using the energy device EnSeal® (Ethicon Endo-Surgery, Cincinnati, OH, USA). Subsequently, the tube was disconnected at the level of the abdominal wall. Adhesions between the ventral aspect of the stomach and liver were removed to ensure optimal placement of the liver retractor. The entire scar capsule of the band was dismantled, considering that the band could have been placed “pars flaccida” or “perigastrically.” The entire band was then placed aside.

The gastric pouch was created from 5 to 6 cm below the esophagogastric junction using an Echelon Flex Endopath 60-mm linear stapler (Ethicon Endo-Surgery, Cincinnati, OH, USA) over a 40 Fr bougie using gold and blue reloads. Whenever possible, we tried to create the pouch above the level of the band. The same stapler was used to construct the gastro-jejunostomy and jejuno-jejunostomy using blue and white reloads, respectively, with equal 100-cm biliopancreatic and alimentary limbs. The stapling defects were closed in two layers using barbed 3/0 V-Loc 180 sutures (Covidien, Mansfield, MA, USA). The staple line in the gastric pouch and remnant stomach was reinforced with continuous seromuscular sutures using the same barbed sutures. All mesenteric defects were closed using 3/0 V-Loc nonabsorbable sutures (Covidien, Mansfield, MA, USA).

### Primary RYGB

A pneumoperitoneum was created using visual entry trocars and a 0° angled lens; five standard ports were used, including three 12-mm ports (for the camera, and right and left working ports) and two 5-mm ports (for liver retraction and the assistant). The pouch was created with the same linear stapler using gold and blue reloads. The lengths of the biliopancreatic and alimentary limbs were equal, 100 cm each, as per revisional RYGB. The gastro-jejunostomy and the jejuno-jejunostomy were performed similarly to revisional RYGB.

All staple lines were reinforced with the same technique used for revisional RYGB. Mesenteric defects were also routinely closed. Intraoperative endoscopy was not routinely performed for primary RYGB. The blue dye leak test was routinely performed for the gastro-jejunostomy. A drain was routinely inserted in the left sub-phrenic space.

## Appendix 2

Table 4

**Table 4:** Lab results pre and postoperative between %EWL < and ≥ 50

After 2 years	Primary	Primary + revision	Primary + revision	Primary + revision	*P* value S1.1–1.0 |1.1–1.2 S1.2–1.0 |1.1–1.3 S1.3–1.0 |1.2–1.3
%EWL ≥ 50 (Strata 1)	RYGB (s1.0) *N* = 398	VBG (s1.1) *N* = 38	Sleeve (s1.2) *N* = 48	Gastric band (s1.3) *N* = 23	
Lab results (mean ± SD)					
Cholesterol (pre-op) (mg/dL)	185.6 ± 60.3	189.6 ± 52.2	169.6 ± 39.6	178.5 ± 45.9	All > 0.273
Cholesterol (post-op) (mg/dL)	161.8 ± 22.4	176.9 ± 45.2	164.5 ± 33.3	168.5 ± 34.9	S 1.0–1.1 = 0.004 All > 0.14
TG (mg/dL) (pre-op)	145.9 ± 35.1	119.1 ± 42.5	115.6 ± 31.8	122.8 ± 32.9	S1.1–1.0 = < 0.01 S1.2–1.0 = < 0.005 S 1.31.0 = < 0.004 Rest > 0.85
TG (mg/dL) (post-op)	132.1 ± 21.6	112.6 ± 35.9	110.5 ± 23.9	116.5 ± 29.8	S1.1–1.0 = < 0.001 S1.2–1.0 = < 0.001 S 1.3–1.0 = < 0.001 Rest > 0.73
LDL (mg/dL) (pre-op)	78.7 ± 22.2	80.5 ± 34.3	74.5 ± 21.5	76.6 ± 12.1	All > 0.62
LDL (mg/dL) (post-op)	70.0 ± 8.9	75.2 ± 27.0	71.1 ± 14.1	72.5 ± 10.3	All > 0.13
HB1ac (mmol/mol) (pre-op)	5.1 ± 1.5	4.8 ± 1.6	5.2 ± 1.5	4.4 ± 0.6	All > 0.17
HB1ac (mmol/mol) (post-op)	4.8 ± 0.6	4.7 ± 1.2	4.6 ± 0.9	4.3 ± 0.7	All > 0.15
Fasting glucose (mg/dL) (pre-op)	100.4 ± 24.1	99.1 ± 18.4	96.4 ± 19.4	94.4 ± 12.5	All > 0.45
Fasting glucose (mg/dL) (post-op)	93.2 ± 12.1	97.9 ± 15.3	92.9 ± 16.7	97.8 ± 14.1	All > 0.07
After 2 years	Primary	Primary + revision	Primary + revision	Primary + revision	*P* value S2.1–2.0 |2.1–2.2 S2.2–2.0 |2.1–2.3 S2.3–2.0 |2.2–2.3
%EWL < 50 (Strata 2)	RYGB (s2.0) *N* = 160	VBG (s2.1) *N* = 128	Sleeve (s2.2) *N* = 32	Gastric band (s2.3) *N* = 69	
Lab results (mean ± SD)					
Cholesterol (mg/dL) (pre-op)	181.9 ± 44.3	186.3 ± 46.1	196.1 ± 48.2	192.6 ± 56.1	All > 0.269
Cholesterol (mg/dL) (post-op)	168.9 ± 31.2	174.4 ± 38.1	176.9 ± 40.3	177.9 ± 44.8	All > 0.292
TG (mg/dL) (pre-op)	138.1 ± 25.6	125.7 ± 40.1	121.5 ± 38.1	122.6 ± 35.2	S 2.1–2.0 = < 0.001 S 2.2–2.0 = < 0.001 S 2.3–2.0 = < 0.001 Rest > 0.92
TG (mg/dL) (post-op)	132.7 ± 21.6	118.7 ± 33.1	115.5 ± 37.4	116.2 ± 32.4	S 2.1–2.0 = < 0.001 S 2.2–2.0 = < 0.001 S 2.3–2.0 = < 0.001 Rest > 0.94
LDL (mg/dL) (pre-op)	72.5 ± 11.3	80.1 ± 29.9	86.2 ± 35.6	75.6 ± 22.1	S 2.1–2.0 = < 0.001 S 2.2–2.0 = < 0.001 Rest > 0.13
LDL (mg/dL) (post-op)	70.8 ± 9.9	74.5 ± 22.9	79.7 ± 31.5	73.9 ± 20.8	S 2.1–2.0 = < 0.008 S 2.2–2.0 = < 0.03 Rest > 0.16
HBa1c (mmol/mol) (pre-op)	4.6 ± 0.72	5.0 ± 1.5	4.5 ± 1.2	5.1 ± 1.6	S 2.1–2.0 = < 0.008 S 2.3–2.0 = < 0.014 Rest > 0.23
HBa1c (mmol/mol) (post-op)	4.5 ± 0.7	4.5 ± 0.9	4.4 ± 0.9	4.7 ± 1.2	All > 0.22
Fasting glucose (mg/dL) (pre-op)	95.9 ± 15.3	97.8 ± 17.5	93.8 ± 13.3	97.7 ± 16.4	All > 0.49
Fasting glucose (mg/dL) (post-op)	94.9 ± 14.2	94.8 ± 15.6	94.3 ± 12.1	96.2 ± 17.9	All > 0.91

*TG* triglyceride, *LDL* low-density lipoprotein, *HBa1c* hemoglobin A1C

## Appendix 3

Table 5

**Table 5:** Associated medical problems pre and postoperative between %EWL ≥ 50 (Strata 1)

After 2 years	Primary	Primary + revision	Primary + revision	Primary + revision	*P* value S1.1–1.0 |1.1–1.2 S1.2–1.0 |1.1–1.3 S1.3–1.0 |1.2–1.3
N (%)	RYGB (s1.0) *N* = 398	VBG (s1.1) *N* = 38	Sleeve (s1.2) *N* = 48	Gastric band (s1.3) *N* = 23	
DM (preoperative)					*p* = 0.326
Absent	313 (78.6)	33 (86.8)	35 (72.9)	23 (100)	
Present	85 (21.4)	5 (13.2)	13 (27.1)	0	
DM (postoperative)					
Free	315 (79.1)	33 (86.8)	35 (72.9)	23 (100)	S1.0 ≤ 0.001
Improved	27 (6.8)	2 (5.3)	7 (14.6)	0	S1.1 = 0.023
Resolution	56 (14.1)	1 (2.6)	1 (2.1)	0	S1.2 = 0.16
Regain	0	2 (5.3)	5 (10.4)	0	S1.3 = 0.9
Hypertension (preoperative)					*P* = 0.322
Absent	290 (72.9)	32 (84.2)	34 (70.8)	19 (82.6)	
Present	108 (27.1)	6 (15.3)	14 (29.1)	4 (17.4)	
Hypertension (postoperative)					
Free	304 (76.4)	32 (84.2)	34 (70.8)	19 (82.6)	
Improved	1 (0.2)	2 (5.3)	5 (10.4)	2 (8.7)	
Resolution	93 (23.4)	3 (7.9)	2 (4.2)	0	
Regain	0	1 (2.6)	7 (14.6)	2 (8.7)	*P* = 0.432
Dyslipidemia (preoperative)					*P* = 0.534
Absent	314 (78.9)	29 (76.3)	42 (87.5)	18 (78.3)	
Present	84 (21.1)	9 (23.7)	6 (12.5)	5 (21.7)	
Dyslipidemia (postoperative)					*P* = 0.324
Free	315 (79.1)	29 (76.3)	42 (87.5)	18 (78.3)	
Improved	19 (4.8)	5 (13.2)	3 (6.25)	2 (8.6)	
Resolution	64 (16.1)	0	0	1 (4.5)	
Regain	0	4 (10.5)	3 (6.25)	2 (8.6)	

## Appendix 4

Table 6

**Table 6:** Associated medical problems pre and postoperative between %EWL < 50 (Strata 2)

After 2 years	Primary	Primary + revision	Primary + revision	Primary + revision	*P* value S2.1–2.0 |2.1–2.2 S2.2–2.0 |2.1–2.3 S2.3–2.0 |2.2–2.3
N (%)	RYGB (s2.0) *N* = 160	VBG (s2.1) *N* = 128	Sleeve (s2.2) *N* = 32	Gastric band (s2.3) *N* = 69	
DM (preoperative)					*P* = 0.331
Absent	151(94.4)	106 (82.8)	28 (87.5)	58 (84.0)	
Present	9 (0.6)	22 (17.2)	4 (12.5)	11 (16.0)	
DM (postoperative)					
Free	151 (94.4)	106 (82.8)	28 (87.5)	58 (84.0)	
Improved	3 (1.8)	10 (7.8)	3 (9.3)	3 (4.3)	
Resolution	6 (3.8)	5 (3.9)	0	0	
Regain	0	7 5.5)	1 (3.2)	8 (11.7)	*P* = 0.314
Hypertension (preoperative)					*P* = 0.322
Absent	123 (76.8)	102 (79.7)	27 (84.3)	60 (86.9)	
Present	37 (23.2)	26 (20.3)	5 (15.7)	9 (13.1)	
Hypertension (postoperative)					*P* = 0.267
Free	125 (78.1)	102 (79.5)	27 (84.3)	60 (86.9)	
Improved	0	10 (7.8)	4 (12.5)	3 (4.3)	
Resolution	35 (21.9)	5 (3.9)	0	2 (2.8)	
Regain	0	11 (8.8)	1 (3.2)	4 (6.0)	
Dyslipidemia (preoperative)					*P* = 0.5342
Absent	127 (79.4)	98 (76.5)	21 (65.6)	54 (78.2)	
Present	33 (20.6)	30 (23.5)	11 (34.4)	15 (21.8)	
Dyslipidemia (postoperative)					*P* = 0.674
Free	127 (79.3)	98 (76.5)	21 (65.6)	54 (78.2)	
Improved	4 (2.5)	13 (10.1)	5 (15.6)	7 (10.1)	
Resolution	29 (18.2)	2 (1.5)	1 (3.2)	1 (1.6)	
Regain	0	15 (11.9)	5 (15.6)	7 (10.1)	
